# Circulating Microvesicles in Association with the NLRP3 Inflammasome in Coronary Thrombi from STEMI Patients

**DOI:** 10.3390/biomedicines10092196

**Published:** 2022-09-05

**Authors:** Vibeke Bratseth, Jostein Nordeng, Ragnhild Helseth, Svein Solheim, Sissel Åkra, Harald Arnesen, Gemma Chiva-Blanch, Ingebjørg Seljeflot

**Affiliations:** 1 Center for Clinical Heart Research, Department of Cardiology, Oslo University Hospital Ullevål, 0424 Oslo, Norway; 2 Institute of Clinical Medicine, Faculty of Medicine, University of Oslo, 0318 Oslo, Norway; 3 Department of Endocrinology and Nutrition, August Pi i Sunyer Biomedical Research Institute-IDIBAPS, Hospital Clinic of Barcelona, 08036 Barcelona, Spain; 4 Spanish Biomedical Research Network in Pathophysiology of Obesity and Nutrition (CIBEROBN), Institute of Salud Carlos III (ISCIII), 28029 Madrid, Spain

**Keywords:** microvesicles, NLRP3 inflammasome, IL-6 signalling pathway, ST-elevation myocardial infarction, Annexin V, immunothrombosis

## Abstract

Microvesicles (MVs) are actively secreted by cells. The NLRP3-inflammasome and the interleukin 6 (IL-6)-pathways are central in cardiovascular disease. Knowledge of how the inflammasome influences the MVs is limited. In a cross-sectional study, we assessed whether MVs in plasma associate with genes encoding inflammasome signalling in coronary thrombi. Moreover, any relationships between inflammasome activation and phosphatidylserine (PS) externalization, determined through Annexin V (AV^+^) labelling, and myocardial injury, assessed by cardiac troponin T (cTnT), were analysed. Intracoronary thrombi and blood samples from STEMI patients (*n* = 33) were investigated. mRNA of NLRP3, caspase-1, interleukin-1β (IL-1β), interleukin-18 (IL-18), IL-6, soluble IL-6-receptor (sIL-6R), and glycoprotein-130 (gp130) were isolated from the thrombi and relatively quantified by RT-PCR. MVs were analysed by flow cytometry. Total AV^+^ MVs, mainly reflecting hypercoagulability, correlated positively to NLRP3 gene expression (r = 0.545, *p* = 0.009). A similar pattern was seen for platelet, endothelial and leukocyte derived MVs, separately. The majority of the MVs were AV^−^ (96%). Total and AV^−^ MVs correlated inversely with IL-1β (r = −0.399 and −0.438, respectively, *p* < 0.05, both) and gp130 (r = −0.457 and −0.502, respectively, *p* < 0.05, both). No correlations between MVs and cTnT were observed. Our findings indicate an association between NLRP3-inflammasome in coronary thrombi and procoagulant AV^+^ MVs in STEMI patients. The inverse relationships between AV^−^ MVs and the gene expression of inflammasome activation may indicate an immuno-dampening role of this subpopulation.

## 1. Introduction

Circulating microvesicles (MVs) are small (0.1–1.0 μm) membrane vesicles actively secreted by almost all cell types [1]. They belong to the umbrella term of extracellular vesicles (EVs), and they contain cytoplasmic material and transmembrane receptors specific for their parent cells. MVs are heterogeneous and essential in several physiological and pathological processes, such as coagulation/thrombosis, inflammation, and intercellular signalling [2,3,4]. Their procoagulant features are attributed to the externalization of phosphatidylserine (PS) and the presence of coagulation factors [5,6]. Recently emerging evidence supports the presence of MVs lacking PS exposure determined through Annexin V (AV^+^) labelling and their significance are unclear [7,8,9].

Inflammation is an important driver of cardiovascular diseases (CVD), both atherosclerosis and acute myocardial infarction (MI) [10]. A cross-talk between inflammation and coagulation, i.e., immunothrombosis, contributes to the progression of CVD [11].

The Nod-Like-Receptor-Protein-3 (NLRP3) inflammasome is a natural part of the innate immune system, and it is activated by various endogenous danger signals abundantly present during atherogenesis [12,13]. The upregulation of NLRP3- and interleukin-1β (IL1β) is enhanced by the binding of pathogen-associated molecular patterns (PAMPs) and damage-associated molecular patterns (DAMPs) to unique receptors, such as Toll-Like-Receptor-4 (TLR4) on activated B cells, inducing the nuclear factor κ-light-chain-enhancer of activated B cells (NF-κB)-mediated transcription [12,14]. Finally, caspase-1 mediates release of the proinflammatory cytokines IL-1β and interleukin-18 (IL-18) and pyroptosis [15]. Inflammasome activation may induce coagulation through pyroptosis and release of tissue factor (TF)^+^ MVs from macrophages [16]. Therefore, exploring the inflammasome and its molecular mechanisms in the manifestation of prothrombotic phenotypes becomes pertinent [17,18].

Another central pathway is the interleukin-6 (IL-6) signalling system. IL-6 exerts pleiotropic effects on inflammation and the immune system. Anti-inflammatory effects are executed via classic signalling where IL-6 binds to the membrane-bound IL-6 receptor (IL-6R), which interacts with the signalling receptor protein glycoprotein 130 (gp130) [19]. In contrast, in trans signalling, IL-6 binds to a soluble form of IL-6R (sIL-6R), activating the membrane-bound gp130 and inducing downstream proinflammatory responses [20]. 

The most common cause of an acute ST-elevation MI (STEMI) is plaque rupture with subsequent thrombus formation, and the essential treatment of these patients is urgent revascularization by thrombolysis or acute percutaneous coronary intervention (PCI). In some cases of large residual thrombus burden, thrombus aspiration may be considered [21] and that creates an opportunity to study the thrombus content closer.

We have previously shown inflammasome-related genes to be upregulated in coronary thrombi from STEMI patients [22]. Growing evidence indicates that inflammasome activity correlates with the enhanced secretion of EVs and the modulation of their protein cargo [23,24]. In the present study we investigated whether circulating MVs and AV^+^, as well as AV^−^, associate with genes encoding inflammasome components (NLRP3, caspase1, IL1-β, and IL-18) and the IL-6 signalling pathway, present in the aspirated thrombi and with corresponding circulating proteins. Our hypothesis was that inflammasome activation present in the occluded thrombi contributes to an accelerated release of MVs and associates with PS externalization on the MVs. In addition, any relationship between the MVs and the degree of myocardial injury as measured by peak cardiac troponin T (cTnT), time from symptoms onset to PCI (ischaemic time) and clinical characteristics were investigated.

## 2. Materials and Methods

### 2.1. Study Population and Sample Preparation

Patients with STEMI (*n* = 33) treated with primary PCI at Oslo University Hospital, Ullevål, Norway, during August 2015 to January 2019 were included in the Thrombus Aspiration in ST-elevation myocardial infarction (TASTI) study. Inclusion criteria were both sexes, age 18–85 years, admission with typical symptoms of acute STEMI in the electrocardiogram (ECG) and treated with primary PCI with thrombus aspiration. STEMI was classified as significant ST-elevation in at least two adjacent ECG-leads. Significant ST-elevation was defined as ≥0.1 mV in the standard leads and precordial lead V4–V6, and ≥0.2 mV in precordial lead V1-V3. Presumed new left bundle branch block or typical posterior infarction pattern, with ST-depressions in lead V1–V4, were assumed as STEMI equivalents. Patients with signs of infection, pulmonary embolism, chronic obstructive pulmonary disease or arrhythmias, abnormal renal or liver function, autoimmune disease, and malignant disease were excluded. The study was approved by the Regional Committee of Medical Research Ethics, in South-Eastern Norway (2015/169) and conducted according to the Declaration of Helsinki. All patients gave their written informed consent. The trial is registered at clinicaltrials.gov with identification number NCT02746822.

Intracoronary thrombi were collected by standard aspiration catheter, washed with saline, snap-frozen in ribonucleic acid (RNA)later solution (Qiagen, Hilden, Germany) and kept frozen at −80 °C for the later isolation of RNA and gene expression analyses. Peripheral blood samples were drawn at the end of the PCI procedure for routine and study-specific analyses. Citrated blood (3.8% sodium citrate) was centrifuged at 2500× *g* × 20 min at 4 °C to prepare platelet-poor plasma (PPP), which was stored at −80 °C until analysis after study completion.

### 2.2. Laboratory Analyses

Gene expression analyses. The details of the gene expression analyses have previously been described [22]. In brief, RNA was isolated from the thrombi using High Pure RNA Tissue Kit with the addition of Proteinase K Solution (Roche Diagnostics GmbH, Mannheim, Germany), stabilized by lysing buffer, and homogenized with a thermomixer (Thermomixer Eppendorf, Eppendorf AG, Hamburg, Germany) and stainless-steel grinding balls (Qiagen GmbH, Hilden, Germany). Real-time PCR was performed on a ViiA™ 7 instrument (Applied Biosystems, Foster City, CA, USA) using TaqMan^®^ Universal PCR Master Mix (P/N 4324018) and the following TaqMan^®^ assays: NLRP3 (Hs00918082_m1), Caspase-1 (Hs00354836_m1), IL-1β (Hs01555410_m1), IL-18 (Hs00155517_m1), IL-6 (Hs00174131_m1), IL-6R (Hs01075664_m1), and gp130 (Hs00174360_m1) (Applied Biosystems). The mRNA levels were relatively quantified using β2-microglobulin (HS99999907_m1) (Applied Biosystems) as house-keeping gene [25] and with a relative quantification (RQ) value of 1.0, the gene analysed was equally expressed as the reference sample.

Circulating levels of inflammatory proteins. Commercially available ELISAs were used for the quantification of circulating levels of IL-6, sIL-6R, gp130 (Quantikine^®^ HS ELISA, R&D Systems^®^, Minneapolis, MN, USA), and IL-18 (MBL, Medical & Biological Laboratories CO., LTD., Nagoya, Japan). The respective inter assay coefficient of variation (CV) in our laboratory were 10.6%, 3.6%, 5.2%, and 2.6%.

Flow cytometric analysis of MVs. The MVs fraction was separated from citrated PPP through a two-step high-speed centrifugation. Five-hundred microliters (µL) frozen plasma samples were quickly thawed for 5 min at 37 °C and gently vortexed. Thereafter, 250 µL (125 µL × 2) were transferred to a new vial and spun at 1300× *g* for 10 min (20 °C) to remove potential clots and debris. Then, 226 µL (113 µL × 2) were collected and centrifuged at 20,000× *g*, for 30 min, to pellet the MVs. The supernatant was removed and the MVs pellets were washed once with citrate-phosphate buffered saline (PBS) solution before a second identical centrifugation step was made. Finally, the MVs pellets were resuspended in 75 µL citrate-PBS. Four-labelled flow cytometry was applied to identify and characterize the MVs. The membrane dye carboxyfluorescein diacetate succinimidyl ester (CFSE) at 100 µM was used to ensure MVs integrity and to discard debris and membrane/cell fragments. For simplicity, CFSE^+^ MVs will be referred to as MVs throughout the manuscript. Furthermore, Annexin V (AV), a cellular protein with relative high affinity for PS and 14 different monoclonal antibodies (mAb) directed against cell-surface molecules for cell origin and activation status, was used and examined in eight different combinations (Supplementary Tables S1 and S2). Briefly, the combinations of mAb labelled with phycoerythrin (PE) and peridinin-chlorophyll-protein (PerCP) were mixed with a working solution (1:10 with distilled water) of Annexin binding buffer (ABB; 0.2 µm sterile filtered 0.1 M Hepes (pH 7.4), 1.4 M NaCl, and 25 mM CaCl_2_ solution) and centrifuged at 13,000× *g* for 5 min (20 °C). Thereafter, the supernatant was transferred to a vial containing allophycocyanin (APC)-conjugated AV and FITC-CFSE and vortexed before 45 µL of each combination was added to a 96-well plate with 5 µL sample and incubated for 20 min at RT in the dark. Then, the samples were diluted with ABB and immediately analysed by using the “Auto Collect” mode on an Accuri C6 flow cytometer (BD, Accuri^®^ Cytometers, Inc., San Diego, CA, USA). The acquisition was set to 2 min/sample with a flow rate at 14 µL/min. Forward scatter (FSC), side scatter (SSC), and fluorescence data were obtained with the settings in the logarithmic scale.

The MVs gate was originally set with the Megamix-Plus FSC, a mix of beads with MVs-equivalent sizes: 0.1, 0.3, 0.5, and 0.9 µm (Biocytex, Marseille, France) (Supplementary Figure S1) and we have later confirmed the gate by acquiring platelet rich plasma (PRP). MVs isolated from the PRP, both unstained and stained with CFSE were analysed to define the CFSE-gate. Fluorescence minus one (FMO) experiments were performed to determine thresholds for positive events and autofluorescence, ensuring specificity. To reduce background noise, buffers were prepared on the same day and filtered through 0.2 µm pore size filters under vacuum. Sperotech 8 (FL1, FL2 and FL3) and 6 (FL4) peak validation beads (BD Biosciences, San Jose, CA, USA) were utilized daily to monitor the performance of the flow cytometer. Data were analysed with BD CSampler software (version 1.0.264.21; Accuri^®^ Cytometers, Inc.). The number of MVs/µL PPP was calculated according to Nieuwland’s formula [26]. The ratio AV^+^/AV^−^ MVs was used as a measure for the degree of PS externalization.

cTnT was measured at the hospital central laboratory by commercial electrochemiluminescence immunoassay (third generation cTnT, Elecsys 2010, Roche, Mannheim, Germany) with a CV of 7%.

### 2.3. Statistical Analyses

Clinical characteristics are given as numbers (proportions) and medians (25th and 75th percentiles), or as otherwise stated. As most of the variables were not normally distributed, non-parametric statistics were used throughout; Mann–Whitney U test for group comparisons and Spearman’s rho for correlation analyses. SPSS version 26 (SPSS Inc., Chicago, IL, USA) was used throughout. Due to the explorative nature of the study, *p*-values ≤ 0.05 were considered statistically significant.

## 3. Results

### 3.1. Clinical Characteristics

The majority of the study population (n = 33) were men (91%) and mean age was 60 years. One third had hypertension (33%), 12% had type 2 diabetes mellitus, 49% were current smokers and the population was slightly overweight (Table 1). Only one had previous coronary artery disease, which was a previous MI, and median time from start of symptoms to PCI was 152 min. The culprit lesion was located in the left anterior descending artery (LAD) in 49% of the patients and median peak cTnT was 3434 µg/L (Table 1). 

### 3.2. Circulating Microvesicles

The levels of total, AV^−^, and AV^+^ MVs/µL PPP, as well as subgroups according to cell type are presented in Table 2. Platelet-derived MVs were the most abundant (CD61^+^) (125.41 (59.60, 250.83)/µL PPP), together with MVs from leukocytes (CD11b^+^) (64.99 (43.32, 100.39)/µL PPP), granulocytes (CD66b^+^) (36.50 (21.29, 102.46)/µL PPP), and endothelial cells (CD31^+^) (35.40 (27.79, 68.45)/µL PPP).

The combinations of two mAbs presented with very low numbers and/or not detectable, hence MVs were characterized by the integrity of the lipid bilayer (CFSE^+^), PS exposure (AV^+^), and single mAb staining.

The majority of the MVs were AV^−^ (96%) and the dataset included one extreme outlier, which has been excluded from further analyses.

### 3.3. Correlations between Circulating Microvesicles and Inflammasome Gene Expression in Thrombi

Among the aspirated thrombi, 30 were adequately analysed for gene expression, and as previously published, we found genes coding for the measured inflammasome-related variables to be present in 76–100% of the thrombi [22].

The bivariate coefficients of correlation between the total levels of MVs, including AV^−^ and AV^+^ MVs and the gene expression of inflammasome-related markers in thrombi, are shown in Table 3.

Total and AV^−^ MVs correlated inversely with IL-1β and gp130 gene expression (all *p* < 0.05), whereas AV^+^ MVs correlated positively with NLRP3 gene expression (r = 0.545, *p* = 0.009).

**Figure 1:**
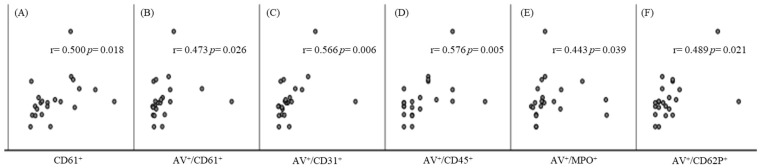
Scatterplots visualizing the correlations between NLRP3 gene expression in thrombi and circulating CD61^+^ MVs (**A**) and AV^+^ MVs derived from platelets (**B**,**F**), leukocytes (**D**), endothelial (**C**), and MPO^+^ (**E**) cells. Abbreviations: MPO—myeloperoxidase.

Regarding MVs derived from different cell types, only those that correlated significantly with the gene expression of inflammasome-related variables in the thrombi are presented in Supplementary Table S3. As shown, mainly AV^+^ MVs derived from platelets, leukocytes/granulocytes, and endothelial cells correlated positively and especially with NLRP3, also visualized in Figure 1.

### 3.4. Correlations between Circulating Microvesicles and Corresponding Circulating Markers 

**Figure 2:**
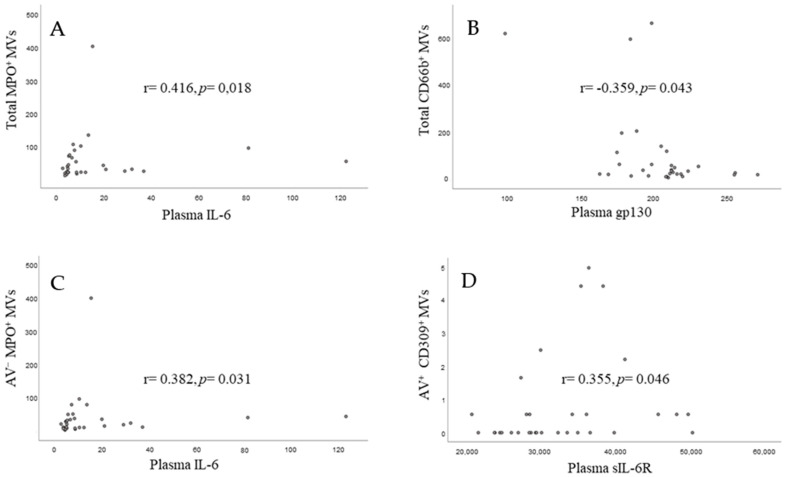
Neutrophil (**A**–**C**)- and endothelial (**D**)-derived MVs and the IL-6 signalling pathway.

The total count of MVs, including AV^−^ and AV^+^, did not correlate significantly with the circulating levels of the corresponding inflammasome variables. However, neutrophil (MPO^+^ and CD66b^+^)- and endothelial (CD309^+^)-derived MVs correlated significantly with the IL-6 signalling pathway, as shown in Figure 2.

### 3.5. PS Externalization on the MVs and the NLRP3 Inflammasome

**Figure 3:**
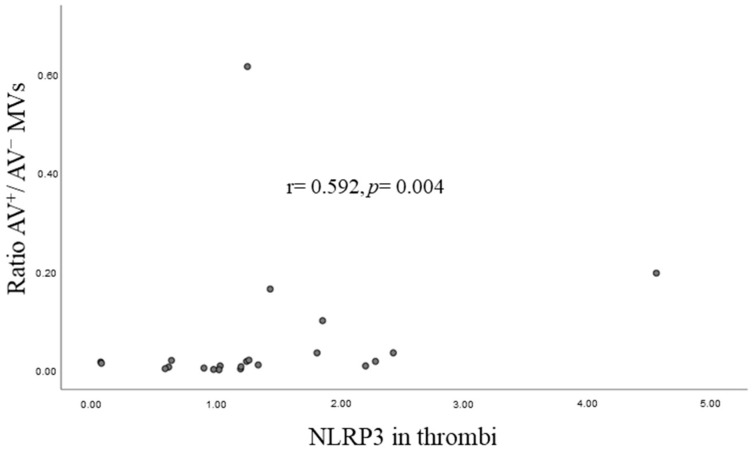
Scatterplot presenting the correlation between the ratio AV^+^/AV^−^ MVs (PS externalization) and NLRP3 gene expression in the thrombi. Abbreviations: PS—Phosphatidylserine.

Bivariate correlation analyses showed that the ratio of AV^+^/AV^−^ MVs correlated positively with NLRP3 gene expression in coronary thrombi (Figure 3) but not to any other investigated genes or circulating markers of the inflammasome, as presented in Table 4.

### 3.6. MVs and Clinical Characteristics 

Total levels of MVs, AV^−^, and AV^+^ were neither correlated to ischaemic time nor peak cTnT, as presented in Table 5; moreover, no correlations within the subgroups of MVs were found (data not shown). Furthermore, no significant differences were observed between current smokers vs. non- and previous smokers and BMI ≥ median vs. BMI < median (27.7 kg/m^2^) (Table 6).

## 4. Discussion

In the current study, we investigated MVs from different cell types, AV^−^ as well as AV^+^, in relation to the NLRP3 inflammasome, the IL-6 signalling pathway, and clinical characteristics in patients with acute STEMI treated with PCI and thrombus aspiration. Our findings indicate an association between procoagulant AV^+^ MVs, the externalization of PS during biogenesis, and the gene expression of NLRP3 in the coronary thrombi. The majority of the MVs were, however, AV^−^, and thus associated inversely with gene expression of inflammasome-related variables in the thrombi. Neutrophil- and endothelial-derived MVs associated with circulating IL-6 signalling markers, though none of the MVs associated with ischaemic time, degree of myocardial injury or clinical characteristics.

The relationship between AV^+^ MVs that mainly derived from platelets and leukocytes/granulocytes, and the expression of the NLRP3 inflammasome in the thrombi, may indicate that AV^+^ MVs are involved in the interplay between the innate immune system, inflammasome activation, and thrombosis [17,18]. During the acute MI, hypoxia–inducible factors, the rupture or erosion of the blood vessel, and activated platelets have been suggested to activate the NLRP3 inflammasome complex via DAMPs, which in turn may lead to a cytokine storm with subsequent cell activation and the release of procoagulant MVs [27]. In an experimental study, the activation of caspase-1 in macrophages was shown to mediate a high surface density of PS via extracellular actin and TF-trafficking along the membrane, generating highly procoagulant MVs [28]. The transcription of TF is induced by the same TLR ligands and the NF-κβ pathway; however, we could not show any associations between MVs carrying TF receptor and the NLRP3 inflammasome in the thrombi.

Growing evidence support a relationship between inflammasome activation, the release of EVs with modified phenotypes, and inflammatory responses [23]. We could also demonstrate inverse associations between total and AV^−^ MVs in endothelial cells and the expression of IL-1*β* and gp130 in the thrombi, suggesting an inhibitory effect of these MVs. Recently, the content and function of inflammasome-induced EVs from macrophages showed that these EVs carried a specific ribonucleic acid (RNA) signature and interferon β. The authors proposed interferon signalling to dampen the NLRP3 inflammatory response in bystander cells, for example by stimulating the endogenous IL-1 receptor antagonist [29].

In the systemic circulation we could demonstrate positive relationships between neutrophil (MPO^+^)- and endothelial (CD309^+^)-derived MVs and IL-6 and IL-6sR, respectively. Neutrophils are attributed a central role in plaque destabilization and their circulating MVs may derive from the plaque due to the procedure or de novo neutrophil activation and propagate inflammation and hypercoagulability by endothelial cell activation and cytokine release [30,31]. Consistent with our results, evidence was recently reported for the acute in vivo release of neutrophil (CD66b^+^ and MPO^+^)-derived MVs after PCI in acute coronary syndrome patients, with blood sampling from the coronary circulation. In addition, they also found a parallel increase of IL-6 in the periphery [32]. Taken together, our results may help to explain the inflammatory response observed in these patients and that some particular MVs may potentially serve as prognostic markers for future events [33,34].

We noted no significant associations between Total, AV^−^, and AV^+^ MVs and ischaemic time, peak cTnT, and clinical variables in our STEMI population. As MVs from platelets are the most abundant, this may be due to the pre-hospital administration of antithrombotics (i.e., aspirin and clopidogrel) as well as the administration of gpIIb/gpIIIa antagonism before the thrombectomy procedure. In addition, the use of heparin during the procedure may have influenced the results. The median ischaemic time was relatively short, and in the main study, no relationships between time from symptoms onset to PCI and the expression of inflammasome-related variables in the thrombi were detected either [22]. Somewhat inconsistent with our results, an inverse relationship between total and cell-specific AV^+^ MVs and ischaemic time has been reported; however, only in non-STEMI and not in STEMI patients [35], suggesting that MVs expression during an acute MI is time-dependent [36,37].

In the current work, we used a conventional flow cytometer that may have influenced the validity of the MVs total count by excluding the smallest MVs [38]. Moreover, we performed a single centrifugation step prior to freezing. However, a second spin was introduced after thawing to remove remnant cells and dust, and all patient samples were processed following the same procedure.

Most of the detected MVs were AV^−^, which was somewhat intriguing, since platelet-derived MVs are the most abundant; however, increasing data support the presence of an AV^−^ population [36,39,40]. Traditionally, circulating MVs have been identified based on their superficial exposure of PS, which may give an incomplete picture of particle shedding. The presence of AV^−^ MVs may also result from the masking of PS by endogenous molecules abundant in the circulation, with concentrations of PS that are too low for AV detection and/or due to other clearance mechanisms [41,42]. Our results are consistent with previous reports demonstrating AV^−^ MVs in patients with procoagulant conditions, such as STEMI, systemic lupus erythematosus (SLE), and type 1 diabetes mellitus [36,39,43].

## 5. Conclusions

Our findings indicate an association between the NLRP3 inflammasome in coronary thrombi and circulating procoagulant AV^+^ MVs in STEMI patients. The AV− MVs were inversely related to gene expression of inflammasome activation, and whether these MVs have an immuno-dampening role, and what their exact functions are, need further investigation.

## Figures and Tables

**Table 1:** Baseline characteristics of the total study population (n = 33). Data are presented as absolute numbers (%) or medians (25th, 75th percentiles) if not otherwise stated.

Baseline Characteristics	
Age, years (mean (range))	60 (38, 83)
Females	3 (9%)
BMI (kg/m^2^)	27.7 (23.4, 28.6)
Hypertension	11 (33%)
T2DM	4 (12%)
Previous CAD	1 (3%)
Current smokers	16 (49%)
Medication:	
ASA	6 (18%)
Plavix	2 (6%)
Anticoagulation	3 (9%)
NOAC	2 (6%)
Betablocker	4 (12%)
AT-II-blocker	5 (15%)
Statins	6 (18%)
Diuretics	4 (12%)
SBP ^a^ (mmHg)	127 (±31)
DBP ^a^ (mmHg)	82 (±19)
Ischaemic time (min)	152 (121, 375)
cTnT after PCI (µg/L)	354 (119, 748)
cTnT peak (µg/L)	3434 (1179, 7652)
CRP (mg/L)	2.71 (0.96, 6.03)
Culprit lesion:	
LAD	16 (49%)
CX	6 (18%)
RCA	11 (33%)
Retrograde flow	12 (36%)
Three-vessel disease	6 (18%)
In-stent thrombus	1 (3%)

^a^ Mean (SD); Abbreviations: BMI—Body Mass Index; T2DM—Type 2 Diabetes Mellitus; CAD—Coronary Artery Disease; ASA—Acetylsalicyclic acid; NOAC—Novel Oral AntiCoagulant; AT-II-blocker—Angiotensin receptor II-blocker; SBP—Systolic Blood Pressure; DBP—Diastolic Blood Pressure; cTnT—cardiac Troponin T; PCI—Percutaneous Coronary Intervention; CRP—C-reactive protein; LAD—Left Anterior Descending Artery; CX—Circumflex artery; RCA—Right Coronary Artery.

**Table 2:** Total levels of MVs and subgroups according to cell type in STEMI patients (n = 32) at the time of PCI. Data are presented as medians (25th and 75th percentiles).

	Total MVs/µL PPP	AV^−^/µL PPP	AV^+^/µL PPP
Total	5112.83 (2807.52, 7139.93)	4917.04 (2660.95, 7112.14)	51.16 (28.07, 92.37)
TF^+^	14.66 (6.91, 26.41)	0.55 (0.00, 1.52)	11.34 (5.67, 25.72)
Platelet			
CD61	125.41 (59.60, 250.83)	79.37 (33.88, 138.69)	25.17 (10.92, 71.21)
CD62P	23.51 (15.49, 38.16)	21.02 (14.38, 34.02)	1.11 (0.55, 2.77)
CD42b	28.76 (15.49, 63.33)	18.53 (10.51, 35.40)	6.36 (1.24, 18.53)
Endothelial			
CD309	5.53 (2.90, 9.40)	4.98 (2.90, 9.26	0.00 (0.00, 0.55)
CD62E	27.10 (19.50, 36.64)	24.34 (17.98, 32.91)	0.00 (0.00, 0.55)
CD31	35.40 (27.79, 68.45)	29.31 (21.43, 46.18)	5.81 (2.35, 11.75)
Leukocytes			
CD14	8.57 (4.98, 16.04)	8.30 (4.98, 14.66)	0.00 (0.00, 0.55)
CD11b	64.99 (43.32, 100.39)	63.33 (45.08, 97.83)	0.55 (0.55, 1.11)
CD62L	2.49 (0.69, 5.91)	2.21 (0.69, 4.84)	0.00 (0.00, 0.55)
CD66b	36.50 (21.29, 102.46)	15.49 (13.0, 34.15)	10.79 (5.81, 43.83)
MPO	30.14 (19.91, 67.89)	21.85 (9.96, 40.79)	9.40 (4.42, 19.91)
CD45	15.49 (11.06, 27.65)	13.27 (9.96, 26.69)	0.55 (0.14, 1.66)
CD15	1.66 (0.55, 3.32)	1.11 (0.55, 3.32)	0.00 (0.00, 0.41)

Abbreviations: MVs—Microvesicles; STEMI—ST-elevation myocardial infarction; PPP—Platelet poor plasma; AV—Annexin V; CD—cluster of differentiation.

**Table 3:** Bivariate correlations between levels of total, AV^−^, and AV^+^ MVs and the gene expression of inflammasome-related markers in thrombi.

		NLRP3	CASPASE-1	IL-1 β	IL-18	IL-6	sIL-6R	gp130
Total	r	−0.322	−0.053	−0.399	−0.170	−0.186	−0.298	−0.457
	*p*	0.143	0.791	0.032	0.428	0.396	0.124	0.019
AV^−^	r	−0.362	−0.062	−0.438	−0.190	−0.129	−0.340	−0.502
	*p*	0.098	0.752	0.017	0.373	0.558	0.076	0.009
AV^+^	r	0.545	−1.06	0.114	0.108	−0.186	0.113	0.003
	*p*	0.009	0.592	0.557	0.615	0.395	0.568	0.988

Abbreviations: NLRP3—Nod-Like-Receptor-Protein-3; IL—Interleukin; s—soluble; R—receptor; gp—Glycoprotein.

**Table 4:** Bivariate correlations between the ratio AV^+^/AV^−^ MVs (PS externalization) and inflammasome gene expression in the thrombi and corresponding circulating markers.

		NLRP3	IL-1β	Caspase-1	IL-18	IL-6	sIL-6R	gp130
Thrombi genes	r	0.592	0.309	−0.018	0.165	−0.005	0.279	0.279
	*p*	0.004 ^a^	0.103	0.927	0.440	0.980	0.151	0.151
Plasma	r				0.073	−0.139	0.034	−0.034
	*p*				0.690	0.449	0.855	0.854

^a^ The correlation was still significant after excluding two extreme outliers: r = 0.538, *p* = 0.014.

**Table 5:** Bivariate correlations between MVs, ischaemic time, and peak cTnT.

		Ischaemic Time	Peak cTnT
Total MVs	r	−0.137	0.208
	*p*	0.454	0.254
AV^−^ MVs	r	−0.096	0.228
	*p*	0.600	0.210
AV^+^ MVs	r	−0.139	−0.007
	*p*	0.448	0.969

**Table 6:** Total, AV^−^, and AV^+^ MVs as related to clinical characteristics in the STEMI population (median (25th and 75th percentiles)).

		Total MVs (µL/PPP)	AV^−^ MVs (µL/PPP)	AV^+^ MVs (µL/PPP)
Smoke ^a^	No smoke (n = 16)	5249.17 (2571.49, 7844.72)	5144.91 (2525.17, 7776.41)	45.77 (24.34, 82.96)
	Current smoker (n = 15)	5068.58 (2976.77, 5841.26)	4122.23 (2712.39, 5310.84)	51.99 (30.42, 95.13)
	*p*-value	0.797	0.621	0.374
BMI ^b^	<median (n = 13)	4850.11 (2751.66, 6594.30)	4822.46 (2600.39, 6564.44)	43.69 (27.10, 68.86)
	≥median (n = 14)	5414.55 (2742.95, 7370.44)	5414.55 (2742.95, 7370.44)	62.09 (25.44, 146.99)
	*p*-value	0.577	0.680	0.452

^a^ n = 31; one patient was lacking smoke history. ^b^ Median BMI was 27.7 (23.4, 28.6) kg/m^2^, n = 27; since five patients were lacking BMI data.

## Data Availability

The data supporting the results of this study are available from the corresponding author upon reasonable request.

## Supplementary Materials

The following supporting information can be downloaded at: www.mdpi.com/article/10.3390/biomedicines10092196/s1; Table S1: Cell molecules for MVs identification and characterization; Table S2: The combinations of mAb together with CFSE and AV; Table S3: Bivariate correlations between MVs from different cell types and inflammasome gene expression in thrombi; and Figure S1: Gate limits for MVs analyses with the Megamix-Plus FSC beads for cytometer settings in MVs analyses.
