# Supplementary material for: Circulating Microvesicles in Association with the NLRP3 Inflammasome in Coronary Thrombi from STEMI Patients
Source: Biomedicines. 2022 Sep 5;10(9):2196. doi: 10.3390/biomedicines10092196 (PMC9496021; doi:10.3390/biomedicines10092196)
Supplement: Supplementary file 1 [file biomedicines-10-02196-s001.zip › biomedicines-1840653-supplementary/Supplementary File 01.09.22.pdf]

**Table S1.** Cell molecules for MVs identification and characterization

| | Alternative name | Expression | Conjugation | Clone | Company |
|---|---|---|---|---|---|
| CFSE | Carboxyfluorescein diacetate succinimidyl ester | Intracellular, membrane dye | FITC | | BD Biosciences |
| Annexin V | Phosphatidylserine (PS) binding protein | Widely expressed | APC | | BD Biosciences |
| **mAb:** | | | | | |
| CD142 | Tissue factor, F3, thromboplastin | Platelet, endothelial and leukocyte activation | PE | NY2 | Nordic Biosite AS |
| **Platelet-related:** | | | | | |
| CD61 | β3-integrin | Platelets | Per-CP | RUU-PL 7F12 | BD Biosciences |
| CD62P | P-selectin | Platelet activation | Per-CP | P.seK02.22 | Diuvita Immunostep |
| CD42b | Glycoprotein 1b, receptor for vWF | Platelet activation | PE | REA 185 | Miltenyi Biotec Norden |
| **Endothelial-related:** | | | | | |
| CD62E | E-selectin (Endothelial-leukocyte adhesion molecule-1 (ELAM-1)) | Endothelial activation | Per-CP | TEA2/1 | Diuvita Immunostep |

| | | | | | |
|---|---|---|---|---|---|
| CD309 | Vascular endothelial growth factor receptor-2 (VEGFR-2) | Endothelial progenitor cells | PE | 89106 | BD Biosciences |
| CD31 | Platelet-endothelial cell adhesion molecule-1 (PECAM-1) | Widely expressed. Platelet endothelial cell adhesion molecule. | Per-CP | TP1/15 | Diuvita Immunostep |
| **Leukocyte-related:** | | | | | |
| CD45 | Leukocyte Common Antigen (LCA) | Leukocytes | Per-CP | HI30 | Diuvita Immunostep |
| CD15 | Lewis X | Granulocytes, monocytes/macrophages | PE | HI98 | Immunotools |
| CD11b | Macrophage-1 Antigen (MAC-1)/ Integrin alpha-M | Monocytes, granulocytes and natural killer cells | Per-CP | DCISI/18 | Diuvita Immunostep |
| CD14 | Lipopolysaccharide (LPS)-receptor | Macrophages, monocytes | PE | M5E2 | BD Biosciences |
| CD62L | L-selectin | Leukocyte activation | PE | LT-TD180 | Immunotools |
| CD66b | Carcinoembryonic antigen-related cell adhesion molecule 8 (CEACAM8) | Granulocyte activation | PE | G10F5 | BD Biosciences |

| | | | | | |
|---|---|---|---|---|---|
| MPO | Myeloperoxidase | Neutrophils (azurophilic granules) | PerCP | 2C7 | Bio-Techne Ltd<br><br>BioNovus Biologicals$^{TM}$ |

Abbreviations: MVs Microvesicles, mAb Monoclonal antibodies, CD Cluster of Differentiation, FITC Fluoresceinisotiocyanat, APC Allophycocyanin, PE Phycoerythrin, PerCP Peridin chlorophyll protein, vWF von Willebrand Factor

**Table S2.** The combinations of mAb together with CFSE and AV

| mAb combination | PE-conjugated mAb | Per-CP-conjugated mAb | CFSE-FITC | AV-APC |
|---|---|---|---|---|
| 1 | CD142 | CD61 | CFSE | APC |
| 2 | CD62L | CD62p | CFSE | APC |
| 3 | CD42b | CD31 | CFSE | APC |
| 4 | CD309 | CD62E | CFSE | APC |
| 5 | CD142 | CD62E | CFSE | APC |
| 6 | CD15 | CD45 | CFSE | APC |
| 7 | CD14 | CD11b | CFSE | APC |
| 8 | CD66b | MPO | CFSE | APC |

Abbreviations: mAb monoclonal antibodies, PE Phycoerythrin, PerCP Peridin chlorophyll protein, CD Cluster of Differentiation, FITC Fluoresceinisotiocyanat, CFSE carboxyfluorescein diacetate succinimidyl ester, AV Annexin V, APC Allophycocyanin

**Table S3.** Bivariate correlations* between MVs from different cell types and inflammasome gene expression in thrombi

| | | NLRP3 | CASPASE-1 | IL-1 β | IL-18 | IL-6 | sIL-6R | GP130 |
|---|---|---|---|---|---|---|---|---|
| **Total** | r | 0.500 | | | | | | |
| CD61 | p | **0.018** | | | | | | |
| CD62E | r | | | | | | | −0.428 |
| | p | | | | | | | **0.029** |
| **AV⁻** | r | | | | | | | −0.488 |
| CD62E | p | | | | | | | **0.011** |
| **AV⁺** | r | 0.473 | | | | | | |
| CD61 | p | **0.026** | | | | | | |
| CD42b | r | 0.559 | | | | | | |
| | p | **0.007** | | | | | | |
| CD31 | r | 0.566 | | | | | | |
| | p | **0.006** | | | | | | |
| CD45 | r | 0.576 | | | | | | |
| | p | **0.005** | | | | | | |

| | | | | | | | | | |
|---|---|---|---|---|---|---|---|---|---|
| $MPO^+$ | r | 0.443 | | | | | | | |
| | p | **0.039** | | | | | | | |
| CD62P | r | 0.489 | | | | | | | |
| | p | **0.021** | | | | | | | |

* Spearmans rho. Only the significant ones are presented.

Abbreviations: MVs Microvesicles, CD Cluster of Differentiation, AV Annexin V, NLRP3 Nod-Like-Receptor-Protein 3, IL Interleukin, sIL-6R, soluble IL-6 receptor, GP glycoprotein, MPO Myeloperoxidase

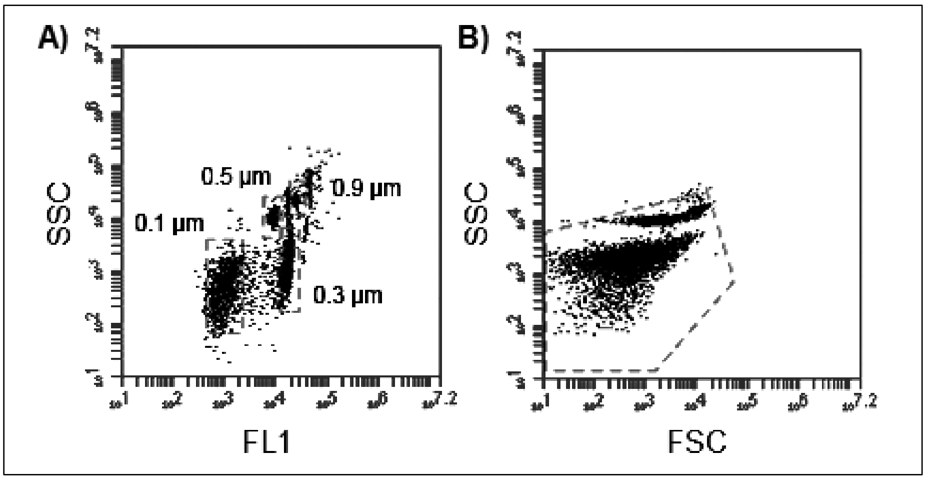


**Figure S1.** Gate limits for MVs analyses with the Megamix-Plus FSC beads for cytometer settings in MVs analyses.

Abbreviations: SSC side scatter, FL1 Fluorescence channel 1, FSC Forward scatter
